# Development of a Single Vial Kit Solution for Radiolabeling of ^68^Ga-DKFZ-PSMA-11 and Its Performance in Prostate Cancer Patients

**DOI:** 10.3390/molecules200814860

**Published:** 2015-08-14

**Authors:** Thomas Ebenhan, Mariza Vorster, Biljana Marjanovic-Painter, Judith Wagener, Janine Suthiram, Moshe Modiselle, Brenda Mokaleng, Jan Rijn Zeevaart, Mike Sathekge

**Affiliations:** 1 University of Pretoria & Steve Biko Academic Hospital, Crn Malherbe and Steve Biko Rd, Pretoria 0001, South Africa; E-Mails: Thomas.ebenhan@gmail.com (T.E.); marizavorster@gmail.com (M.V.); janine.suthiram@necsa.co.za (J.S.); modisellemoshe@yahoo.co.uk (M.M.); tshelom@gmail.com (B.M.); 2 School of Health Sciences, Catalysis and Peptide Research Unit, E-Block 6th Floor, Westville Campus, University Road, Westville, Durban 3630, South Africa; 3 The South African Nuclear Energy Corporation (Necsa), Building P1600, Radiochemistry, Pelindaba, Brits 0240, South Africa; E-Mails: biljana.marjanovic-painter@necsa.co.za (B.M.-P.); judith.wagener@necsa.co.za (J.W.); 4 Department of Science and Technology, Preclinical Drug Development Platform, North West University, 11 Hoffman St, Potchefstroom 2520, South Africa; E-Mail: janrijn.zeevaart@necsa.co.za

**Keywords:** PSMA, prostate cancer, PET/CT, ^68^Ga-HBED-CC-(Ahx)Lys-NH-CO-NH-Glu, ^68^Ga-DKFZ-PSMA-11, ^68^Ga-PSMA^HBED^

## Abstract

Prostate-specific membrane antigen (PSMA), a type II glycoprotein, is highly expressed in almost all prostate cancers. By playing such a universal role in the disease, PSMA provides a target for diagnostic imaging of prostate cancer using positron emission tomography/computed tomography (PET/CT). The PSMA-targeting ligand Glu-NH-CO-NH-Lys-(Ahx)-HBED-CC (DKFZ-PSMA-11) has superior imaging properties and allows for highly-specific complexation of the generator-based radioisotope Gallium-68 (^68^Ga). However, only module-based radiolabeling procedures are currently available. This study intended to develop a single vial kit solution to radiolabel buffered DKFZ-PSMA-11 with ^68^Ga. A ^68^Ge/^68^Ga-generator was utilized to yield ^68^GaCl_3_ and major aspects of the kit development were assessed, such as radiolabeling performance, quality assurance, and stability. The final product was injected into patients with prostate cancer for PET/CT imaging and the kit performance was evaluated on the basis of the expected biodistribution, lesion detection, and dose optimization. Kits containing 5 nmol DKFZ-PSMA-11 showed rapid, quantitative ^68^Ga-complexation and all quality measurements met the release criteria for human application. The increased precursor content did not compromise the ability of ^68^Ga-DKFZ-PSMA-11 PET/CT to detect primary prostate cancer and its advanced lymphatic- and metastatic lesions. The ^68^Ga-DKFZ-PSMA-11 kit is a robust, ready-to-use diagnostic agent in prostate cancer with high diagnostic performance.

## 1. Introduction

Prostate cancer (PC) is the most commonly diagnosed cancer in men globally and is the second leading cause of death from malignancy among men in USA and other countries [1]. Currently, the available conventional diagnostic procedures, such as preventive blood diagnostics of tumour marker levels like prostate specific antigen (PSA), or inconvenient methods such as the digital rectal prostate examination or transrectal ultrasound (TRUS), are debatable. Non-invasive methods include sonographic Doppler techniques [2], computed tomography (CT), or magnetic resonance imaging (MRI) [3] of the abdomen and pelvis. These procedures frequently reveal PC but are lacking accuracy for detection of PC staging as well as in recurrent PC [4]. A hybrid imaging approach whereby CT is combined with positron emission tomography (PET) using Fluorine-18 fluorodeoxyglucose (FDG) has more significance, as the glucose metabolism can be instrumented to detect numerous malignancies. However, FDG-PET bears limitations towards the detection and localization of slow-growing primary prostate cancer and initial staging of disease with a tumour uptake level that can overlap with those in normal tissue and benign prostatic hyperplasia [5].

Published reports have highlighted the advantages of using ^11^C- or ^18^F-radiolabeled derivatives of acetate or choline, ^18^F-labeled testosterone-derivatives as a ligand to androgen receptors, as well as PET radiotracers targeting prostate-specific membrane antigen (PSMA), prostate stem cell antigen- or gastrin-releasing peptide receptor [6,7,8]. ^11^C-Choline-PET/CT and ^18^F-Choline-PET/CT have been performed for years, however, in a considerable number of cases the metabolism of the tumor has not increased enough for the choline analogs to detect PC, especially in recurrent PC [9,10,11]—confirming the need for a more universal imaging agent. In the assessment of metastatic PC, it is becoming increasingly clear that ligands targeting PSMA may be a superior alternative to the aforementioned prostate cancer PET imaging agents. It is a type II membrane glycoprotein that is significantly overexpressed during all stages of the androgen-insensitive or the metastatic cancer of the prostate compared to other PSMA-expressing tissues, such as kidney, proximal small intestine, or salivary glands [12,13,14,15]. In 2008 M. Pomper and colleagues initiated the evaluation of radio-halogenated, technetium- and rhenium-labeled urea-based inhibitors of PSMA [16,17] to develop more potent imaging agents for prostate cancer. The PSMA-inhibiting peptide-based motif “glutamate-urea-lysin” (Glu-NH-CO-NH-Lys-(Ahx)) was discovered as a novel pharmacological entity. PSMA ligand derivatives were subsequently conjugated to 1,4,7,10-tetraazacyclododecane-1,4,7,10-tetraacetic acid (DOTA) to allow for ^68^Ga-complexation, but notable concerns about the structural changes were addressed [18], as the DOTA macrocycle in the immediate vicinity decreased the binding affinity [19]. Alternatively, the novel and seldom used acyclic chelator agent *N*,*N*′-bis-[2-hydroxy-5-(carboxyethyl)benzyl]ethylenediamine-*N*,*N*′-diacetic acid (HBED-CC) was introduced to allow interaction with the hydrophobic part of the S1-subunit binding site of the PSMA protein and to facilitate rapid radiolabeling with metal-radioisotopes such as Gallium-68 at ambient temperatures. To date, a clinical pilot investigation with this ^68^Ga-labeled PSMA derivative (Glu-NH-CO-NH-Lys-(Ahx)-(^68^Ga)Ga(HBED-CC)) further denoted as ^68^Ga-DKFZ-PSMA-11) suggested that it detects PC relapses and metastases with higher contrast as compared to ^18^F-labeled choline [20]. The conjugation of HBED-CC enabled the research group to produce highly-specific activities of ^68^Ga-DKFZ-PSMA-11 [21] and clinical experience show that the degree of accuracy of ^68^Ga-DKFZ-PSMA-11 PET/CT will have a huge impact on the management of patients with PC and will address an important unmet need in this field [22].

From a medical point of view, compared to ^18^F-FDG, ^68^Ga allows shorter image acquisition, which has significant financial implications and demonstrates a more cost-effective approach with immediate benefit for patient care. It should also be noted that the ^68^Ga half-life of 67.6 min often matches well with the pharmacokinetic of peptides and other structures, which makes it an attractive labelling option for novel diagnostic applications [23,24]. Particularly from an economic viewpoint, using generator-based PET-radiopharmaceuticals like ^68^Ga has advantages over radioisotopes such as ^11^C, which are produced in a cyclotron ^68^Ga be conveniently yielded daily and GMP-compliant using mild acidic conditions (*i.e.*, 0.1 to 1 M HCl) and immediately used for radiolabeling.

Recently, examples for kit formulation strategies using single vial productions have arisen, such as for ^68^Ga-radiolabeling of DOTA-peptides [25], NODAGA-conjugated compounds [26], and buffered citrate [27]. ^68^Ga-DKFZ-PSMA-11 PET/CT will inevitably be a sensitive tool to support clinical trials and patient care using state-of-the-art diagnostic technologies and will significantly advance diagnosis and treatment management of patients with recurrent PC. To the best of our knowledge only elaborate cassette- and module-based radiolabeling techniques have been published [21]. In view of the suitability of the precursor we set out to develop a single vial kit radiolabeling procedure for ^68^Ga-DKFZ-PSMA-11.

We report here the formulation of a freeze-dried kit containing DKFZ-PSMA-11 and sodium acetate trihydrate. We critically assessed the following aspects concerning kit development: radiolabeling with ^68^Ga eluted from a certified generator, performance, safety, quality, sterility, and stability. Changes that were made to manufacture the DKFZ-PSMA-11 kit included an increase in the precursor content. The final product (having passed all necessary quality control requirements) was injected into patients with PC at various stages of the disease prior to PET/CT imaging. Kit performance was evaluated based on the expected biodistribution, lesion detection, and dose optimization.

## 2. Results and Discussion

### 2.1. ^68^Ge/^68^Ga Generator

1.85 GBq loaded ^68^Ge/^68^Ga-generators were utilized to yield 1.55 GBq ± 0.44 GBq of ^68^Ga-activity, eluted as a 2–3 mL batch. Routinely, 1.0 mL generator eluate (457 MBq ± 210 MBq (*n* = 21)) was added to the DKFZ-PSMA-11 for labeling. Thus, two time-lagged radiosyntheses could be performed from one generator elution facilitating personalized administration (alternatively the full 2–3 mL batch could be used with an upscale protocol). Decayed total generator eluates and ^68^Ga-DKFZ-PSMA-11 samples were routinely measured for residual ^68^Ge. The maximum ^68^Ge amount in the total eluate was 0.00074%. The average amount of ^68^Ge found in the final product solution was 0.000057% ± 0.000015% (*n* = 8)). These levels were well below the limits outlined for ^68^Ge-breakthrough (0.001% according to the Phar. Eur. 8.0). The contents of co-eluted ionic metal impurities as analyzed by ICP-OES was highest for the 246-day-old generator (13.7 ppm); this level was calculated below the limit of 20 ppm for the final product for all tested metals (Al^3+^, Sn^2+^, Fe^3+^, Cu^2+^ and Zn^2+^).

### 2.2. Preliminary Assessment of the Radiolabeling Parameters

**Figure 1:**
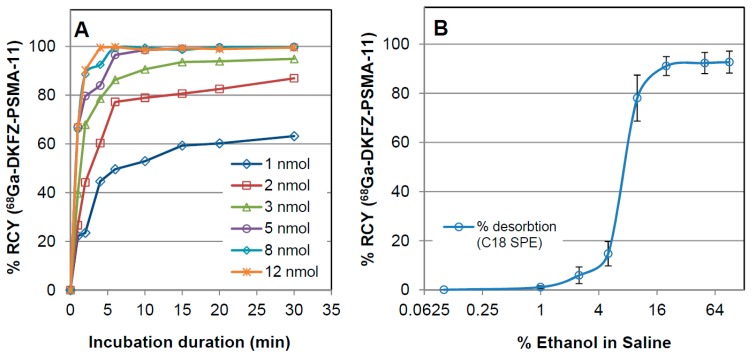
Results from the preliminary assessment of ^68^Ga-DKFZ-PSMA-11 radiolabeling for studying: (**A**) precursor molarity as a function of incubation duration and (**B**) rising ethanol concentration required to desorb the purified product from a C18-SepPak light cartridge unit. The % RCY and % recovery of ^68^Ga-DKFZ-PSMA-11 are displayed (determination of percentage activity of the tracer peak using ITLC). Samples were incubated at RT at pH 4.0–4.5. Mean values (±sem) of three independent experiments are displayed (error bars in A representing sem of 3.7%–12.4% are voided for more transparent presentation).

Prior to the kit formulation, certain parameters were optimized to achieve quantitative complexation of ^68^Ga to DKFZ-PSMA-11. The relevant results are summarized in Figure 1A, showing that under moderate acidic conditions a minimum of 5 nmol DKFZ-PSMA-11 was required in combination with 10 min incubation at RT to warrant a 95%–100% complexation of ^68^Ga. Trace amounts of uncomplexed ^68^Ga were determined with either HPLC or ITLC. Using 15%–25% ethanolic saline solution allowed recovering 84%–95% ^68^Ga-DKFZ-PSMA-11 (Figure 1B) with C18 purification. Vortex stirring action caused no significant increase of the radiochemical yield (*p* = 0.338) or shortening of the incubation time, but is advised to be carried out as described (Material and Methods). An assessment of the radiolabeling depending on changes of pH, incubation temperature, and the choice of buffering solution was voided for this study. Ambient temperature labeling was a prerequisite for the kit formulation and former studies by Eder *et al.* suggested the optimal pH of 4.2 [19]; all crude radiolabeling solutions met the pH range of 4.0–4.5.

### 2.3. In-House Kit Vial Formulation of DKFZ-PSMA-11

The 5 nmol DKFZ-PSMA-11 were buffered with sodium acetate. The kit pellet was presented in a homogeneous-solid powder form, thus, no pellet bulking agents were added to the kit. After supplementing the 1.0 mL ^68^Ga (0.6 N HCl) to the kit vial, the pellet dissolved rapidly to yield a clear particle-free solution. Table 1 summarizes the pre-release tests in comparison to the outcome of the ^68^Ga-DKFZ-PSMA-11 kit-based radiosynthesis, indicating that all quality control tests met the prerequisite criteria.

**Table 1:** Overview of quality control tests compared to the release criteria for safe administration of ^68^Ga-DKFZ-PSMA-11 to humans.

Quality Control Test	Specification	Test Results
Eluate fraction yield (MBq)	≥300 MBq/1 mL	332–1039
^68^Ge breakthrough (total eluate batch 10 mL)	≤0.001% over 9 months	max: 0.00074% mean: 0.0003% ± 0.0001%
Cationic impurities (Zn, Fe, Cu, Sn, Al)	≤50 ppm/1 mL	pass
Product yield (MBq)	≥200 MBq/1 mL	310 ± 52
Visual inspection	Clear colourless, particle-free	pass
Radiochemical identity ITLC	R*_f_* ^68^_Ga-DKFZ-PSMA-11_ = 0.75 ± 0.2	0.73–0.77
Radiochemical identity HPLC	Retention time = 5.3 ± 0.5 min	5.18–5.58
Chemical identity HPLC_(UV214nm)_	Retention time = 4.9 ± 0.5 min	4.83–5.08
Radiochemical purity	≥95%	99.6
pH for injection	physiological (6.0–7.6)	6.5–7.0
Sterile filter integrity	≥3.5 bar	5.9 ± 0.9 (*n* = 7)
Radionuclide identity	67.7 ± 5 min	65.1–69.8 (*n* = 6)
Residual ^68^Germanium (2–5 mL)	≤0.001%	0.000057 ± 0.000015
Sterility	Sterile (fungal/anaerobe/aerobe)	pass
Total product endotoxins	max: 20 EU	pass

**Figure 2:**
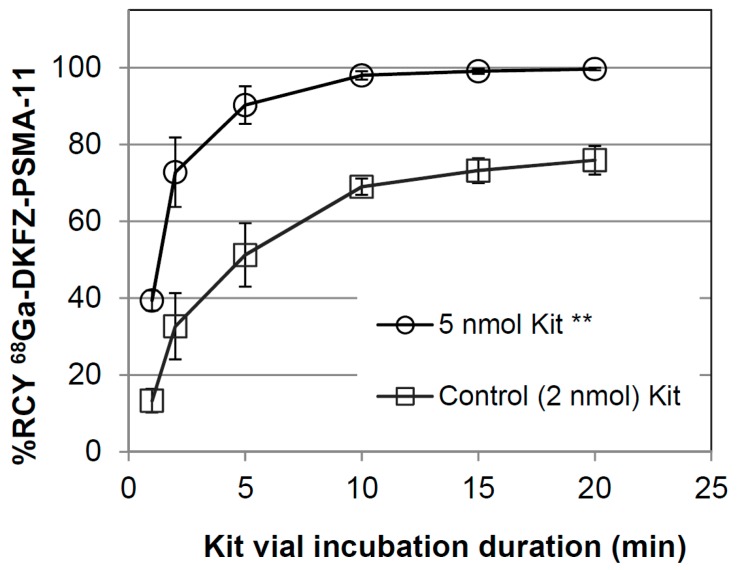
Kit radiolabeling of ^68^Ga-DKFZ-PSMA-11 (Stage 2) as a function of incubation duration (ITLC-analysis mobile phase: Methanol/Saline 20:80 (*v*/*v*)). No purification was carried out post radiolabeling. Mean values (±sem) are displayed. ** Student *t*-tests returned a *p* value ≤ 0.01, for the % RCY of 5 nmol DKFZ-PSMA-11-containing kits (*n* = 4) *vs.* the control (*n* = 3).

**Figure 3:**
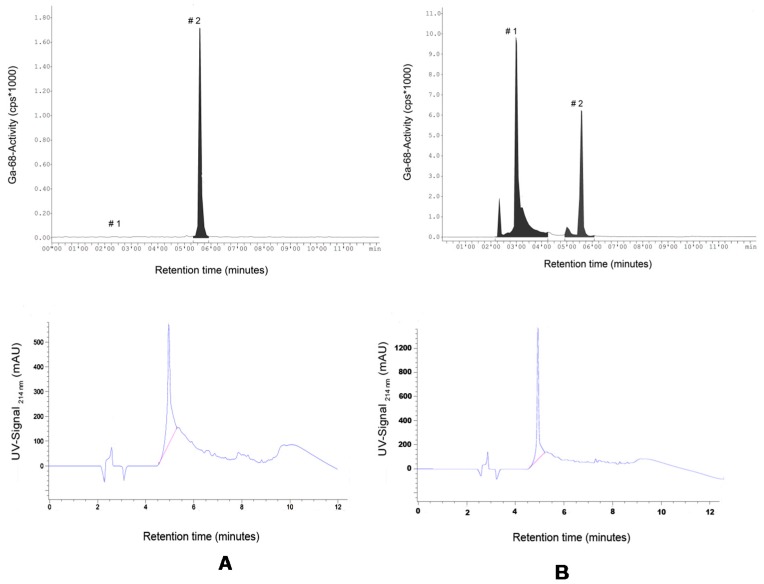
Exemplary HPLC chromatograms as recovered from the radioactivity channel (**top panel**) detecting (#1) free ^68^Ga and (#2) ^68^Ga-DKFZ-PSMA-11. Bottom panel showing DKFZ-PSMA-11 UV-signal detected simultaneously (λ: 214 nm). Samples were analyzed: (**A**) immediately after adding ^68^Ga-activity; and (**B**) after 20 min incubation duration.

This study considered 28 kit based radiosyntheses. Eighteen kits were handled during the investigation of the purification step, here called Stage 1 (Stage 1, see Section 3.5.) and, building on those results, 10 additional kits were applied in Stage 2 where a true one-vial-one-step-radiolabeling approach was explored (Stage 2, see Section 3.5.). The average duration from generator elution to sterile dispensing of ^68^Ga-DKFZ-PSMA was 41 ± 8 min (*n* = 12, phase 1) and 25 ± 4 min (*n* = 5, phase 2, *p* = 0.068) providing up to 800 MBq (calculated yield of two staggered synthesis) of product for injection and for quality-control purposes. The % recovery of ^68^Ga-DKFZ-PSMA-11 using 20% ethanolic saline solution in Stage 1 was 78% ± 13% (*n* = 14). The final product was supplied in 8–10 mL with an average product yield range of 300–650 MBq for imaging purposes. In 16 out of 18 DKFZ-PSMA-11 kits (89%), the amount of uncomplexed ^68^Ga was calculated ≤2.44%, whereas 2 kit radiosyntheses showed uncomplexed ^68^Ga-levels of 6.2% and 6.9% (C18 SepPak purification was applied to all 18 radiosyntheses in phase 1). The % RCP after purification was calculated ≥98.9% (*n* = 13). The results achieved during the kit investigation (Stage 1) were a prerequisite to performing radiolabeling, voiding the purification step (Stage 2). At a pH value of 4.0 the kits containing 5 nmol DKFZ-PSMA-11 complexed the ^68^Ga significantly better (*p* < 0.01) than the reference kit vials containing 2 nmol. The kit preparation of DKFZ-PSMA-11 did not compromise the radiolabeling parameters (Figure 2). HPLC-analysis showed free ^68^Ga-release from the C18-column at *ca.* 2–3 min and compound retention until 5.58 min. The UV-signal intensity for the compound identification was found at slightly earlier times (4.8–5.1 min) due to the consecutive alignment of the radio-HPLC detector (Figure 3 A,B). The DKFZ-PSMA-11 kits radiolabeled during phase 2 showed a % RCP of 96.8%–99.9% (*n* = 7) after 20 min incubation at RT (*p* > 0.01). Both radiolabeling procedures caused a 3%–16% loss of radioactivity to glass vial surfaces and disposal material such as the C18 cartridge. In light of the achieved results, calculations were made to achieve further optimization for a resourceful and economical protocol. The decay-corrected % RCY was very high for both methods applied, amounting to 87% ± 5% and 90% ± 3% (*p* = 0.198) for Stage 1 and Stage 2, respectively. On the basis of the presented results, 0.5 mL of the nine-months-old generator eluted ^68^Ga (~240 MBq) would suffice as starting material to yield an appropriate single patient dose of 150–160 MBq after 25 min production. The ^68^Ga eluted from a 30-days-old generator could be used in 0.25 mL aliquots/patient using kit adjustments, respectively. This might be the key aspect to an economic commercialization of the kit technique. Alternatively, if the PET imaging capacity will allow timely image acquisition of multiple patients, the kit vial can be adjusted to perform an upscaled radiolabeling procedure that serves for 4–5 injections. However, it should be noted that GMP complaint quality controls should be repeated accordingly.

### 2.4. Quality Assessment of the ^68^Ga-DKFZ-PSMA-11 Kit

#### 2.4.1. Appearance and Sterility

Optical inspection showed a clear, colourless product solution, which was free of particles or sediments. Decayed batches of ^68^Ga-DKFZ-PSMA-11 were successfully tested free of anaerobe and aerobe bacteria, as well as fungal growth. Moreover, none of the sterile filters failed the integrity (bubble point burst) test ≥ 62 psi (*ca.* 4.1 bar).

#### 2.4.2. Radionuclidic Identity and pH Value

The ^68^Ga-samples tested for half-life (radionuclidic) identification yielded results of 66.8 ± 2.9 min and could be differentiated from ^68^Ge-samples (265.7 ± 4.9 min; *n* = 4). The pH value was *ca.* 4.0 for the reaction mixture and in physiological range for all product solutions for injection.

#### 2.4.3. Radiochemical Stability

The radiochemical integrity of the DKFZ-PSMA-11-complexed ^68^Ga was found to be >98% in 20% ethanolic saline solution at 37 °C over the 240-min duration observed (*n* = 3). Free ^68^Ga-levels were found 0.3% ± 0.05%, 0.7% ± 0.18%, and 1.9% ± 0.31% for 60, 120, and 240 min, respectively. This high thermodynamic stability would allow for multi-patient production and resourceful optimization of the kit production as addressed earlier.

#### 2.4.4. Long-Term Storage and Radiolabeling Reproducibility

**Figure 4:**
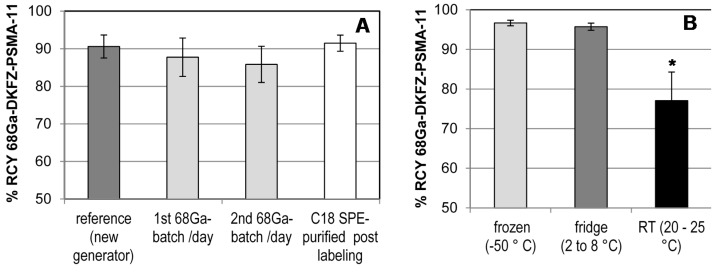
^68^Ga-DKFZ-PSMA-11 kit radiolabeling performance: (**A**) effect of purity of the eluted ^68^Ga; and (**B**) long-term storage at different temperatures. Kit contents were labeled at RT for 15 min at pH 4.0–4.5 and analysed using radio-ITLC. Mean values (± sem) of two to nine independent experiments are displayed. * Statistical significance tests returned *p* values ≤0.05 for the % RCY of DKFZ-PSMA-11 kits incubated at RT *vs.* % RCY of frozen and/or cooled kit vials.

There is limited information from former studies about the longitudinal performance of the iThemba LABS generator. As shown in Figure 4A, the reformulated kits give consistently high radiochemical yields when tested with 1.0 mL radiogallium eluted from a freshly manufactured generator (dark-grey column). No significant difference in the % RCY was found when the DKFZ-PSMA-11-kits were labeled with 1.0 mL eluate of the 1st batch, 2nd batch (light gray columns) of a nine-months-old generator or purified post labeling by SepPack C18 light (open-white column). The %RCY amounted to 90% ± 3.4%, 88% ± 5.1%, 86% ± 4.8%, and 94% ± 1.3%, for the aforementioned batches, respectively. Particular consideration was taken regarding the potential influence of the kit storage condition The DKFZ-PSMA-11 kit performance due to altered long-term storage (Figure 4B) showed % RCY of 97% ± 0.7%, 96% ± 0.9%, and 77% ± 7.2 % (*p* = 0.008) for vials that were frozen at −50 °C, cooled at 2 °C to 8 °C, and placed at RT, respectively. To date the frozen kits (4 months) perform stable radiosyntheses of DKFZ-PSMA-11 and the stability is monitored continuously. The single vial kit approach for the tracer preparation satisfies the necessity of a standardized pharmaceutical product with controlled quality and wide availability.

### 2.5. Clinical PET/CT—^68^Ga-DKFZ-PSMA-11 Kit Performance in Prostate Cancer Patients

**Figure 5:**
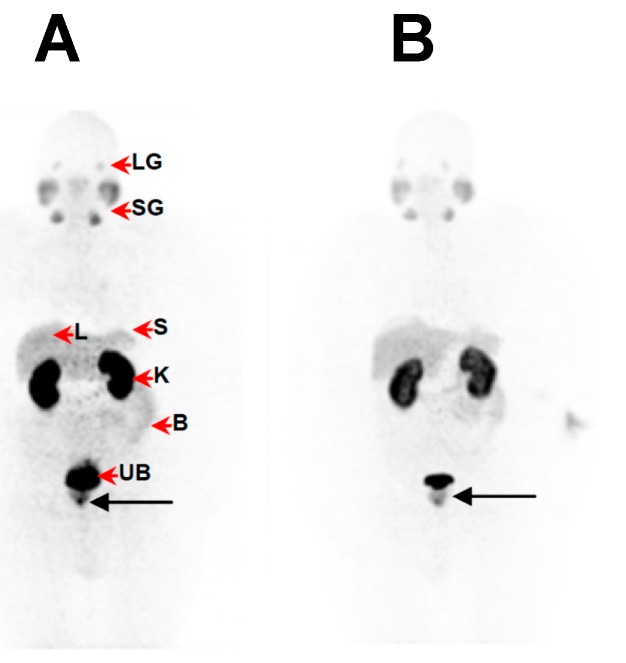
^68^Ga-DKFZ-PSMA-11-PET images showing pathology of primary prostate cancer (**black arrow**) in a 63-yr-old patient (weight = 125 kg; PSA = 146 μg/L; ^99m^Tc-MDP-SPECT was negative for lesions) at 60 min following administration of (**A**) a low dose of ^68^Ga-DKFZ-PSMA-11 (97 MBq) and (**B**) a high dose of ^68^Ga-DKFZ-PSMA-11 (325 MBq), respectively. Images were obtained on a Siemens Biograph 40 PET/CT scanner and displayed in anterior projection. The normal bio-distribution (**red arrow heads**) consists of uptake in the lacrimal glands (LG), salivary glands (SG), liver (L) and spleen (S), minimal bowel excretion (B), and main excretion via the kidneys (K) into the urinary bladder (UB).

**Figure 6:**
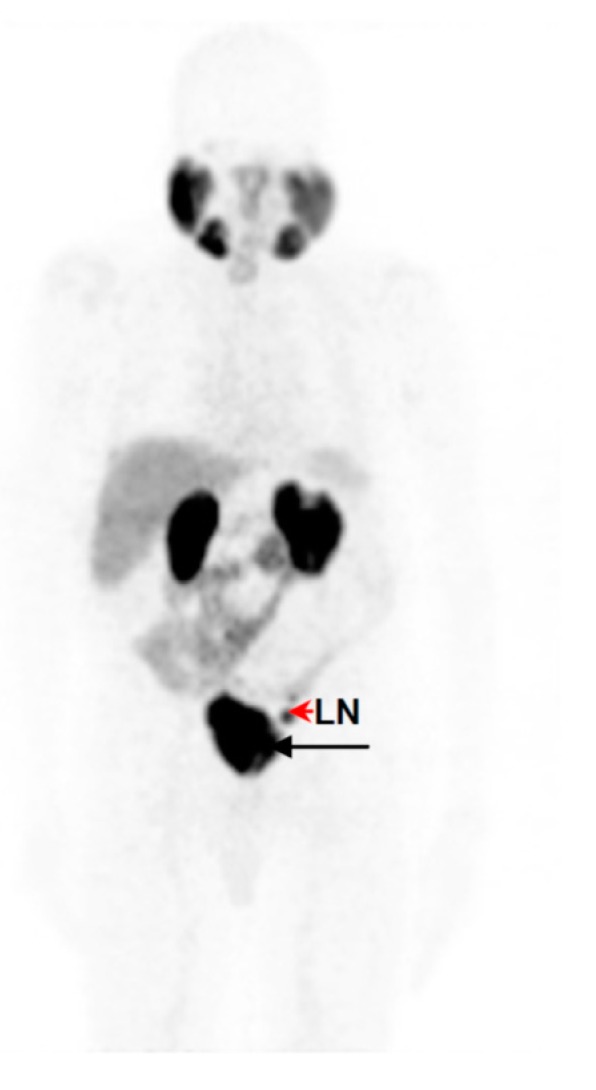
PET image displayed in anterior view of ^68^Ga-DKFZ-PSMA-11 in a 92-yr-old male with limited disease at presentation (weight = 65 kg; history of rising PSA levels post orchidectomy for prostate; no prior ^18^F-FDG-PET or ^99m^Tc-MDP-SPECT carried out). Prostate cancer is demonstrated (**black arrow**) including an intense accumulation of ^68^Ga-DKFZ-PSMA-11 in the left internal iliac node (**red arrow head**) at 60 min post-injection.

**Figure 7:**
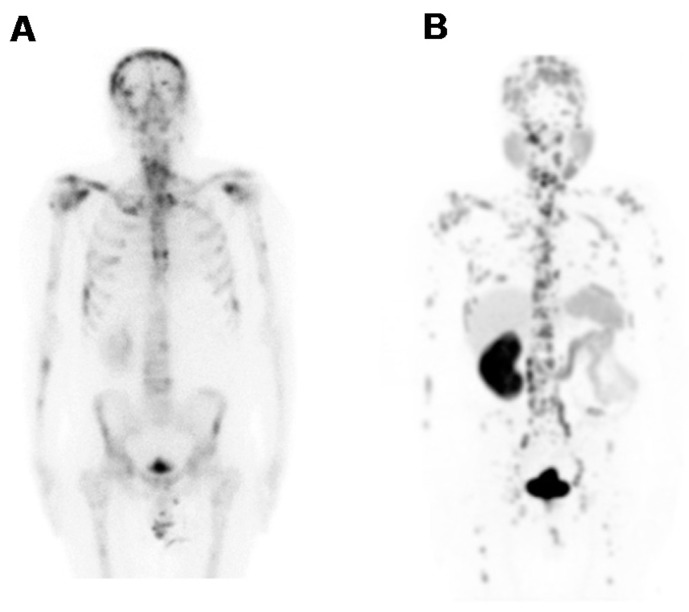
Whole body image projections of a 63-yr-old patient presenting with advanced disease (weight = 77 kg; PSA = 291 µg/L) (**A**) ^99m^Tc-MDP-SPECT was positive for bone metastases; (**B**) PET/CT imaging detected widely distributed skeletal and visceral metastases at 60 min after injection of a low dose of ^68^Ga-DKFZ-PSMA-11 (70 MBq). Images were obtained on a Siemens Biograph 40 PET/CT scanner and are displayed in anterior projection, showing multiple skeletal and soft tissue lesions.

Fifteen male patients (up to 92 yr-old, history of rising PSA-levels, with and without ^18^F-FDG-PET or ^99m^Tc-MDP-SPECT (technetium-99m methylene diphosphonate/single photon emission computed tomography) scan prior to this imaging study) were considered for studying the kit performance in various patients to address localization of primary tumours, lymph node involvement and metastasis. Figure 5 demonstrates the image findings in a patient with limited disease at presentation (age: 63-years-old; weight: 125 kg; PSA = 146 μg/L; ^99m^Tc-MDP-SPECT negative for lesions). The organ-distribution of ^68^Ga-DKFZ-PSMA-11 consists of uptake in expected organs such as in the lacrimal glands, salivary glands, liver and spleen, minimal bowel excretion, and main excretion via the kidneys into the urinary bladder. The image quality and the ability to delineate the tumour mass can be considered comparable when using a low dose (Figure 5A) SUV_max_ = 27.3)) instead of a high dose (Figure 5B) SUV_max_ = 23.7)) in the same patient. This is an example how a dose of 97 MBq has sufficiently detected the primary lesion without significantly compromising the image quality. Figure 6 is a typical example of a patient with a limited history of the disease at presentation (age: 92-yr-old; weight = 65 kg; history of rising PSA levels post-surgery (orchidectomy)). No ^18^F-FDG-PET or ^99m^Tc-MDP-SPECT was carried out prior to ^68^Ga-DKFZ-PSMA-11-PET, which showed an advanced disease state with an involvement of lymph nodes in the pelvis area (SUV_max_ = 18.3); the low dose of 44.4 MBq injected localized the pathologic tissues adequately. In Figure 7, a patient had a surgical history of bilateral orchidectomy and left nephrectomy. He represents a typical example of advanced prostate cancer (age: 63-yr-old; weight: 77 kg; PSA = 291 μg/L) with a positive ^99m^Tc-MDP-SPECT scan for bone metastases (Figure 7A). ^68^Ga-DKFZ-PSMA-11 PET/CT confirmed prostate cancer recurrence (SUV_max_ = 21.6) including multiple soft tissue and bone metastatic lesions with SUV_max_ values ranging from 8.6–22.6 (Figure 7B).

### 2.6. Discussion

Nuclear medical PET/CT-diagnostics using ^18^F-FDG, or ^11^C or^18^F labeled derivatives of choline and acetate is based on cellular glucose accumulation or tumour proliferation rate, which is found to be low in most forms of PC [28]. However, alternative PET imaging with ligands targeting receptors such as PSMA that are overexpressed independently from tumour proliferation seem superior also because PSMA ligands exhibit the ability to be internalized by receptor-mediated endocytosis [29]. This binding, in combination with a suitable radioisotope, allows for highly selective PET imaging with intense tumour-to-background ratios as a result of enhanced tumour cell retention of the tracer molecule. In 2009 scientists described the pseudo-peptide structure Glutamine-Urea-Lysin to be a PSMA-targeting inhibitor that clears rapidly from the circulation, leading to images with clear contrast [30,31]. As ^68^Ga was envisaged as the preferred radioisotope, the first challenge occurring was to successfully complex it with DOTA without hampering the targeting abilities. The limited binding of DOTA-PSMA was overcome by the conjugation of Glutamine-Urea-Lysin to the hetero-bifunctional chelator HBED-CC [21,32] to form DKFZ-PSMA-11, which warranted efficient radiolabeling with ^68^Ga at ambient temperature. The high specificity of DKFZ-PSMA-11 to complex ^68^Ga led us to set up a convenient kit labeling procedure. Additionally, PC imaging using the kit manufacturing technique is tested for its performance in PC patients.

To our knowledge, this is the first report on this novel single vial kit assessment involving a SnO_2_-based ^68^Ge/^68^Ga generator presenting ^68^Ga-DKFZ-PSMA-11-PET images with high lesion-to-background ratios. The major challenge was to address an appropriate precursor concentration and radiolabel it with ^68^Ga while meeting all safety and purity requisites, without compromising the straightforward radiolabeling. In the past five years, ^68^Ge/^68^Ga-generators have emerged as a reliable, source for daily ^68^Ga, possibly detaching PET-radiopharmaceutical development from a cyclotron environment. We managed to utilise a fractionated batch of ^68^Ga that was eluted from a commercially available SnO_2_-based generator (IDB Holland, Netherlands). The amount of elutable ^68^Ga-activity over the generator’s nine-month-life span was excellent and the breakthrough of ^68^Ge and co-eluted metal impurities were of no concern for these radiopharmaceutical productions (Table 1). If the DKFZ-PSMA-11-peptide-labeling formulation can be supplied in a GMP-compliant kit form in analogy to all the conventional ^99m^Tc-radiotracers, it can be made available to all the PET/CT facilities in South Africa and beyond.

The concept of utilizing PET-kits was first addressed by E. Deutsch 1993 in the *Journal of Nuclear Medicine*, where he suggests that in order to satisfy the governmental regulatory and manufacturing requirements one significant approach would entail the development of a family of ^68^Ga-radiopharmaceuticals that can be prepared from cold kits and a ^68^Ge/^68^Ga-generator [33]. Nowadays, we have singular kit vial solutions becoming an asset to PET radiopharmaceutical research, as exemplified by a universal technique to radiolabel proteins [26] or macro-aggregated albumin [34] with ^68^Ga. In 2013 we have successfully reported a procedure of a one-step-aseptic technique to radiolabel ACD-A kits with ^68^Ga for imaging of tuberculosis [27,35]. Many problems concerning the regulation and manufacture of PET radiopharmaceuticals can be alleviated in their future development. However, careful, critical attention to detail and rigorous quality-assurance protocols are essential if complex radiolabeling procedures are to be successfully embedded at a hospital radiopharmacy [36].

The kit-derived ^68^Ga-DKFZ-PSMA-11 was injected into patients diagnosed with PC. PET/CT images demonstrated the expected bio-distribution with the most intense tracer accumulation noted in the kidneys and salivary glands. The lacrimal glands, liver, spleen, and the small and large intestines showed moderate-to-low uptake (Figure 5), which confirmed what other groups have demonstrated [14,37]. Moreover, our amended approach to work with an increased mass of DKFZ-PSMA-11 (to achieve quantitative ^68^Ga-complexation) did not hamper the pharmacological ability of the molecule; a clear delineation of the tumour mass through the PET scan was determined, even with less than 100 MBq administered, as a result of the high specific activity. The low injected doses in this study (*n* = 6) were lower than doses administered to study dosimetric aspects of ^68^Ga-DKFZ-PSMA-11 [38]. These doses exhibit less radiation burden (effective dose of 3 mSv), delivering organ doses that are comparable to (kidneys), or lower than, those delivered by ^18^F-FDG. Following low-dose tracer administration, we were able to detect primary tumours (Figure 5) and early metastatic disease with pelvic lymph node involvement (Figure 6), as well as widespread metastatic disease (Figure 7). It should be noted that this clinical investigation is merely a performance test to address the radiopharmaceutical amendments in the production according to GMP and GCP. This study cannot motivate the application of low doses routinely for this tracer, but it might be interesting to implement this objective in a larger scaled investigation. Low dose tracer administrations might bear the risk of overlooking critical lesions or leaving malignant loci undetected post therapy. If accurately investigated, the use of lower injected doses offers significant advantages; it helps to reduce patient radiation exposure, while allowing for the optimization of costs and patient throughput. Cyclotron-independent PET tracers also allow for the possibility of PET/CT centres in rural areas.

Based on these results, the performance of the ^68^Ga-DKFZ-PSMA-11 kit appears similar to published findings with ^68^Ga-DKFZ-PSMA-11, which were produced in a module- or cassette-like procedure [21,22]. We have also reported a case study where ^68^Ga-DKFZ-PSMA-11 PET/CT was successfully imaging metastatic breast cancer [39] and the tracer was also found to accumulate in metastatic renal cell carcinoma [40].

The ^68^Ga-DKFZ-PSMA-11 kit is a safe and useful, ready-to-use diagnostic agent in PC with high diagnostic performance. A multi-patient dose can be produced and dispensed in less than 30 min at RT, featuring high thermodynamic stability, and a high degree of reproducibility and robustness towards the storage environment and the quality of the eluted radiogallium. The simplicity of the method provides a highly convenient and easy-to-integrate ^68^Ga-tool to tracer production in the hospital radiopharmacy. Providing an immaculate generator performance, this simplified technique makes post-purification obsolete and usable by radiography personnel. The latter approach may be suitable for implementation of other ^68^Ga-radiopharmaceuticals for a more elegant way of translational research.

## 3. Material and Methods

### 3.1. Chemicals and Materials 

If not stated otherwise, only pharmacological-grade solvents were used in the procedures. DKFZ-PSMA-11 was purchased from ABX advanced biochemical compounds (Biomedizinische Forschungsreagenzien GmbH, Radeberg, Germany) in GMP-compliant grade, supplied as trifluoroacetate salt. A 30% solution of ultrapure grade hydrochloric acid (HCl), trifluoroacetic acid and methanol were purchased from Fluka Analytical (Steinheim, Germany). High-performance liquid chromatography (HPLC) grade water (resistivity = 18.2 MΩ cm) was produced in-house by a Simplicity 185 Millipore system (Cambridge, MA, USA). All other solvents were purchased in at least HPLC grade from Sigma Aldrich (Steinheim, Germany). Certified sterile pyrogen-free sealed borosilicate glass vials (5–30 mL) were provided by NTP Radioisotopes (Pty) Ltd. (Pelindaba, South Africa) and were utilized for kit production, generator elution, and sterile saline dispension. Silica gel ITLC paper was purchased from Agilent Technologies (Forest Lakes, CA, USA). Sterile filters were obtained from Millipore (Millipore, New York, NY, USA).

### 3.2. ^68^Ge/^68^Ga Generator

^68^Ga (89%; EC β^+^ max. 1.9 MeV) was yielded from two consecutive tin-dioxide-based ^68^Ge/^68^Ga generators (iThemba LABS, Somerset West, South Africa). The eluate fractionation and purification was carried out as previously reported [27]. A Jelco 22G × 1 polymer catheter (Smiths Medical, Croydon, South Africa) was utilized to warrant metal-free radiogallium transfer. Routinely, inductively coupled plasma optical emission spectroscopy (ICP-OES) was carried out to detect trace metal impurities. The levels of co-eluted ^68^Ge were routinely surveyed in the total generator eluate and in retained ^68^Ga-DKFZ-PSMA-11 solutions. After at least 48 h, ^68^Ge was measured indirectly in a CRC 25 ionization chamber (Capintec Inc., Ramsey, NJ, USA), by detecting radiogallium that was generated *in situ* by ^68^Ge impurities. ^68^Ge samples of known activity were used as references. Low ^68^Ge-levels were detected using a single probe well counter (Biodex Inc., Shirley, New York, NY, USA) or a calibrated gamma spectrometer as previously described [27]. The percentage ^68^Ge was calculated by as follows:

(1) $$68\text{Ge}-\text{impurity }\left(\text{\%}\right)=\frac{68\text{Ge}-\text{activity }\left(\text{Bq}\right)}{68\text{Ga}-\text{activity }\left(\text{Bq}\right)}\;\times 100\text{\%}$$

### 3.3. Preliminary Assessment of Radiolabeling Parameters

In order to achieve the highest labeling efficiency in optimal time, the following labeling parameters were optimized: DKZF-PSMA-11 molarity given a constant ^68^Ga pH value (buffered with sodium acetate trihydrate to yield pH 4.0–4.5) and incubation duration (with or without applying vortex stirring of the sample). Therefore, 1 mL of the ^68^Ga eluate was pre-mixed into vials containing 1–12 nmol DKFZ-PSMA-11 and 98 mg sodium acetate trihydrate salt and incubated at room temperature (RT) up to 30 min; one set of samples underwent vortex stirring action every 5 min for 15 s. At 2, 4, 6, 10, 15, 20 and 30 min, 4 µL per sample was extracted for analysis. The labeling efficiency for all crude samples was determined by ITLC as described. Potential impurities and un-chelated ^68^Ga [41] were purified using Sep-Pak light C18 solid-phase-extraction (Waters, Eschborn, Germany).

### 3.4. In-House Kit Vial Formulation of DKFZ-PSMA-11

Sterile kit vials were produced at Radiochemistry; The South African Nuclear Energy Corporation, using 10 mL vials provided by NTP Radioisotopes. A stock solution of 50 nmol/mL DKFZ-PSMA-11 (resultant mass ^68^Ga-DKFZ-PSMA-11: 5.07 µg (MW_DKFZ-PSMA-11_ = 947.0 g/mol + MW_68Ga_ = 69.7 g/mol) was prepared by dissolving the peptide in Millipore water. Aliquots of 100 µL of the peptide stock solution were mixed with 98 mg sodium acetate trihydrate and vortexed vigorously to yield a homogeneous, gel-like consistency. Alternatively, 5 nmol DKFZ-PSMA-11 were supplemented with 250 µL of 392 mg/mL sodium acetate trihydrate solution and vortexed. The kit vials were immediately placed in an ultra-low freezer (Bio-Freezer, Thermo Fisher Scientific, Waltham, MA, USA) for a minimum of four h (−50 °C) and subsequently transferred to the Alpha 1–5 laboratory freeze dryer (Christ, Osterode am Harz, Germany) where lyophilisation was carried out overnight under Argon atmosphere at 0.05 mbar. The vials were sealed and routinely stored at 2–8 °C.

### 3.5. ^68^Ga Radiolabeling of DKFZ-PSMA-11 Kits

Eighteen kit vials containing 5 nmol buffered DKFZ-PSMA-11 were mixed with 1 mL ^68^GaCl_3_ solution and allowed to incubate at RT for 15 min with gentle vortexing for at least 15 s in five-min intervals. Thereafter the radiolabeling was carried as following: Stage 1 involves the kit investigation phase where the need for a purification step was studied by passing the reaction mixture through a SepPack C18 light as published [42], recovering the product with ethanolic saline solution. Stage 2 involves a true one-vial-one-step-radiolabeling approach followed by supplementing the kit vial with 1.5 mL of 2.5 M sodium acetate trihydrate and 3 mL of saline, to yield a physiological pH. Before dispensing of ^68^Ga-DKFZ-PSMA-11, the solution was run through a 0.22 µm membrane using a low protein-binding filter. An aliquot of >2 mL was retained for further quality assessment of the kits. Radiochemical purity (% RCP) was determined by gradient radio-high-pressure-liquid-chromatography (HPLC) and the decay-corrected radiochemically recovered yield (% RCY) was determined by radio-thin layer chromatography (ITLC) as optimized from previously described procedures [21,43]. A reverse-phase HPLC column (Zorbax SB C18, 4.6 mm × 250 mm × 5 µm; Agilent Technologies, CA, USA) with a linear A–B gradient (0% B to 100% B in 6 min) at a flow rate of 1 mL/min was utilized for quantification analysis. Solvent A consisted of 0.1% aqueous trifluoroacetic acid (TFA) and solvent B was 0.1% TFA in acetonitrile. The HPLC system (Agilent 1200 series HPLC instrument coupled to 6100 Quadruple MS detector, Agilent Technologies Inc., Wilmington, DE, USA), equipped with a diode array detector (DAD) and radioactive detector (Gina Star, Raytest, Straubenhardt, Germany) measured UV absorbance at 214 nm. For ITLC quantification silica gel impregnated chromatographic paper (1 × 10 cm, Agilent Technologies, Forrest Lake, CA, USA) was employed as the stationary phase. The paper was spotted with reaction mixture, dried and exposed for 5–8 min to the mobile phase (Saline/MeOH 80:20 *v*/*v*). The chromatograms were visualized by ITLC radio-chromatography imaging (VSC-201, Veenstra Ind., Oldenzaal, The Netherlands) using a gamma radiation detector (Scionix 25B25/1.5-E2, Bunnik, The Netherlands). The obtained chromatograms allowed for peak identification and performing “area under the curve” analysis for percentage quantification (Genie2000 software, Veenstra Ind., Oldenzaal, The Netherlands). Free radiogallium remained close to the baseline (R*_f_*: 0.05) whereas the ^68^Ga-DKFZ-PSMA-11 peak was detected at R*_f_* of 0.8–0.95.

### 3.6. Quality Assessment of the ^68^Ga-DKFZ-PSMA-11 Kit

Assessment of the quality of the cold or radiolabeled kits included sterility, pH value of the ^68^Ga reaction mixture and product solutions, percentage radiolabeling efficiency (% LE), radiochemical stability, radionuclidic identity and labeling reproducibility with different generator eluate purities, and storage stability.

#### 3.6.1. Appearance and Sterility

After optical and light microscopic inspection of particles and change of colour, aliquots of the sterile filtered sample were analysed by the NHLS microbiological laboratory at Steve Biko Academic Hospital for bacterial growth of aerobe, anaerobe, and fungal species as carried out previously [27]. Sterile filters were tested for pressure integrity (bubble point test).

#### 3.6.2. Radionuclidic Identity and pH Value

Radionuclidic identity was measured by 120 min decay analysis for determination of ^68^Ga half-life using a CRC 25 ionization chamber (Capintec Inc., Ramsey, NJ, USA). The pH value was assessed using a narrow range pH paper (pH Fix 0-7, Macherey-Nagel, Düren, Germany) technique.

#### 3.6.3. Radiochemical Stability

As multi-patient doses can potentially be achieved from an upscale radiopharmaceutical production the stability of ^68^Ga-DKFZ-PSMA-11 was evaluated over three h of incubation at 37 °C and subsequent analysis for potential free ^68^Ga recurrence using radio-ITLC.

#### 3.6.4. Long-Term Storage and Radiolabeling Reproducibility

Verification of the kit shelf-life was carried out considering two aspects A) storage stability (aliquots for the kit formulation were kept at either −50 °C, 2–8 °C, or at room temperature for 30–60 days prior to radiolabeling and B) robustness towards purity changes of the generator-eluted ^68^GaCl_3_: kits contents were mixed with equal ^68^Ga-batches yielded from a freshly-manufactured generator and compared with those of an outdated generator: (1) a routinely eluted generator batch, (2) the consecutive batch of the same day yielded >4 h later, and (3) a C18-purified ^68^Ga-DKFZ-PSMA-11 batch, respectively. Radiolabeling was carried out according to the above mentioned protocol. The % RCY and % RCP was determined by HPLC and ITLC as described earlier. 

### 3.7. Clinical PET/CT—^68^Ga-DKFZ-PSMA-11 Kit Performance in Prostate Cancer Patients

The study was conducted in accordance with the Declaration of Helsinki. The University of Pretoria’s Research Ethics Committee granted approval for this study and written or verbal consent was obtained from each participant prior to tracer injection. Patients with histologically confirmed PC were included with referral indications for initial staging, restaging or suspected recurrence.

#### Image Acquisition, Reconstruction and Analysis

Image acquisition and reconstruction was carried out as previously reported [27]. Briefly, no special patient preparation was required and all patients were imaged on a Siemens Biograph 40-slice PET/CT scanner according to standard protocol. Intravenous contrast was injected unless a contra-indication existed and all patients were imaged from vortex to mid-thigh at 60 min post-injection of low doses of ^68^Ga-DKFZ-PSMA-11 (44-126 MBq). Images were independently analysed by two trained physicians determining the maximum standard uptake values (SUV_max_) in lesions or targeted organs.

### 3.8. Statistical Analysis

If necessary, data was normalized by a log10 transformation before statistical analysis. If not stated otherwise, analytical datasets were expressed as mean and standard error of mean (sem). Dependency between two parameters was analysed by the Spearman correlation to provide the correlation coefficient (*r*). Significance of two mean values was calculated by *Student-t-test* (paired and unpaired comparison). For all statistical tests, the level of significance (*p*) was set at <0.05 (two-tailed) where * *p* < 0.05, ** *p* < 0.01, *** *p* < 0.001 *vs.* references or controls.

## 4. Conclusions

We managed to produce ^68^Ga-DKFZ-PSMA-11 from a freeze-dried one-vial kit that meets all QC criteria for PET imaging. An efficient technique for the routine preparation of ^68^Ga-DKFZ-PSMA-11 was presented, showing highly-specific, reproducible radiolabeling that accommodated the acidic conditions needed to elute ^68^Ga from a SnO_2_-based ^68^Ge/^68^Ga generator. Up to 800 MBq highly pure (>98%) ^68^Ga-DKFZ-PSMA-11 was provided for patient administration within 20–30 min, and the localization of primary PC and lymph node involvement, as well as advanced metastatic PC scenarios, seemed not to be compromised by the kit-manufacturing protocol. Moreover, ^68^Ga-DKFZ-PSMA-11 PET/CT is desirable for an effective stratification of patients undergoing theranostic radioligand therapy with ^177^Lu-labeled PSMA-ligands.
